# Improved Gene Targeting through Cell Cycle Synchronization

**DOI:** 10.1371/journal.pone.0133434

**Published:** 2015-07-20

**Authors:** Vasiliki Tsakraklides, Elena Brevnova, Gregory Stephanopoulos, A. Joe Shaw

**Affiliations:** 1 Total New Energies, Emeryville, California, United States of America; 2 Novogy Inc., Cambridge, Massachusetts, United States of America; 3 Department of Chemical Engineering, Massachusetts Institute of Technology, Cambridge, Massachusetts, United States of America; CNR, ITALY

## Abstract

Gene targeting is a challenge in organisms where non-homologous end-joining is the predominant form of recombination. We show that cell division cycle synchronization can be applied to significantly increase the rate of homologous recombination during transformation. Using hydroxyurea-mediated cell cycle arrest, we obtained improved gene targeting rates in *Yarrowia lipolytica*, *Arxula adeninivorans*, *Saccharomyces cerevisiae*, *Kluyveromyces lactis* and *Pichia pastoris* demonstrating the broad applicability of the method. Hydroxyurea treatment enriches for S-phase cells that are active in homologous recombination and enables previously unattainable genomic modifications.

## Introduction

Integration of a DNA fragment in a host genome requires the action of a double-strand break (DSB) repair mechanism [1, 2]. DSBs caused by environmental factors or natural cellular processes are common and the two most prominent pathways for their repair are non-homologous end-joining (NHEJ) and homologous recombination (HR). Both pathways are highly conserved, suggesting that DSB repair proceeds through nearly universal biological mechanisms [3]. When a cell is transformed with a linear DNA molecule, NHEJ results in random integration of the exogenous DNA into the genome. Such integrations can be useful for the expression of a gene encoded by the introduced DNA. In contrast, HR targets recombination to a homologous locus [4]. Targeted recombination is essential in manipulating genomic loci (e.g. gene targeting) or extra-chromosomal DNA (e.g. recombination cloning). NHEJ and HR coexist in most cells but the balance in activity between them varies among species and cell types. One species with a high ratio of NHEJ-to-HR activity is *Yarrowia lipolytica*, an industrially important non-conventional yeast that has been used for the production of single cell oil and protein [5–7]. In *Y*. *lipolytica* cells transformed with linear DNA, NHEJ prevails resulting in random integration of the construct in the genome regardless of any chromosomal homology. Very few HR-based events occur and the number of transformants that must be screened to obtain strains with targeted integrations can be prohibitively large. For example, we have been unable to obtain gene deletions by targeted integration in the wild-type *Y*. *lipolytica* strain YB-392 (NRRL, ARS Culture Collection) used in our laboratory despite screening hundreds of transformants (Table 1 and data not shown).

**Table 1:** Effect of HU treatment on gene targeting efficiency. Transformants of *Y*. *lipolytica*, *A*. *adeninivorans*, *S*. *cerevisiae*, *P*. *pastoris and K*. *lactis* untreated or pretreated with HU were screened to distinguish random and targeted integration events. The percentage of gene targeting is shown and the number of total transformants screened is included in parentheses. Targeted genes are listed by their systematic names (*Y*. *lipolytica*, *S*. *cerevisiae*, *P*. *pastoris K*. *lactis*) or GenBank accession numbers (*A*. *adeninivorans*). The Fisher’s exact test hypergeometric probability for each individual experiment is tabulated in the last column.

Organism	Target gene	Marker	Targeting homology length		Transformation condition		Fisher’s exact test hypergeometric probability
			upstream	downstream	Untreated cells	HU-treated cells	
					% gene targeting (total transformants screened)	% gene targeting (total transformants screened)	
*Y*. *lipolytica*	YALI0D17534	*hph*	40 bp	39 bp	0% (48)	4% (48)	2.47 x 10^−1^
*Y*. *lipolytica*	YALI0D17534	*hph*	40 bp	39 bp	0% (96)	9% (96)	1.61 x 10^−3^
*Y*. *lipolytica*	YALI0D17534	*hph*	40 bp	39 bp	0% (96)	5% (96)	2.96 x 10^−2^
*Y*. *lipolytica*	YALI0B13970	*hph*	50 bp	40 bp	0% (99)	15% (48)	2.89 x 10^−4^
*A*. *adeninivorans*	KM409710	*hph*	39 bp	37 bp	6% (72)	30% (96)	2.93 x 10^−5^
*A*. *adeninivorans*	KM409711	*hph*	39 bp	37 bp	8% (96)	33% (96)	1.19 x 10^−5^
*A*. *adeninivorans*	KM409712	*hph*	39 bp	40 bp	7% (96)	26% (96)	3.13 x 10^−4^
*A*. *adeninivorans*	KM409713	*hph*	39 bp	40 bp	21% (95)	64% (95)	1.02 x 10^−9^
*A*. *adeninivorans*	KM409714	*hph*	39 bp	40 bp	6% (96)	48% (96)	1.68 x 10^−11^
*S*. *cerevisiae*	YOR128C	*nat*	50 bp	50 bp	6% (639)	17% (258)	4.04 x 10^−07^
*P*. *pastoris*	PAS_chr3_0085	*nat*	50 bp	50 bp	1.6% (374)	5.4% (279)	5.01 x 10^−3^
*K*. *lactis*	KLLA0E02685	*prom-nat-term*	50 bp	50 bp	12% (123)	28% (155)	7.04 x 10^−4^
*K*. *lactis*	KLLA0E02685	*nat*	50 bp	50 bp	79% (89)	97% (831)	1.47 x 10^−9^

For the last 10 years, efforts to improve gene targeting efficiency have focused on genetically abolishing the NHEJ pathway. This approach has been successful in improving HR frequency in *Y*. *lipolytica* [8, 9] and other fungi [10–20], as well as bacteria [21], plants [22–24] and animal cell lines [25–27], but suffers from a number of drawbacks. The method is limited to a specific genetic background (a NHEJ-deficient strain) that may be difficult to obtain as it involves gene deletion or mutation in an organism where gene targeting is challenging. A sequenced and annotated genome is required to identify potential NHEJ targets and these targets have not been equally successful at increasing HR in the studies referenced above. Finally, mutation in the highly conserved DSB repair pathways compromises the cell’s ability to maintain genome integrity. Mutations may be lethal [25] while viable strains can be unstable and suffer from sensitivity to DNA damage and increased mutation rates [9, 23, 28, 29]. These are undesirable traits in industrial strains and there is a need for a method of gene targeting that avoids these pitfalls.

In the present study, we describe a method that reversibly alters the HR-to-NHEJ ratio during cell transformation while maintaining both DSB repair pathways genetically intact. We have taken advantage of a natural and well-conserved oscillation in HR and NHEJ activity during the cell division cycle to significantly increase the frequency of targeted integration. In wild-type cells, the choice between double-strand break repair pathways is influenced by the phase of the cell cycle [3, 30]. In *S*. *cerevisiae*, NHEJ is the preferred pathway for DSB repair in G1, whereas HR is the predominant pathway in S/G2 phase when sister chromatids are available as templates for repair [31, 32]. We hypothesized that increasing the population of S-phase cells during transformation could have a significant effect on the relative frequency of targeted and random integration events. Hydroxyurea-mediated cell cycle arrest was chosen from among a number of cell cycle synchronization protocols [33] because of its ease of use, wide applicability and reversible effect. Hydroxyurea (HU) is a potent inhibitor of ribonucleotide reductase [34–36]. By lowering the levels of dNTPs available for DNA synthesis, HU triggers a Mec1p-dependent signaling cascade that activates the intra-S-phase checkpoint thus resulting in reversible S-phase arrest within a single cell cycle. HU has been applied in the study of cell cycle biology in eukaryotic cells and has been shown to induce recombination in *S*. *cerevisiae* [37]. We show that HU-treatment prior to transformation enables or enhances gene targeting in multiple yeast strains.

## Results

**Fig 1:**
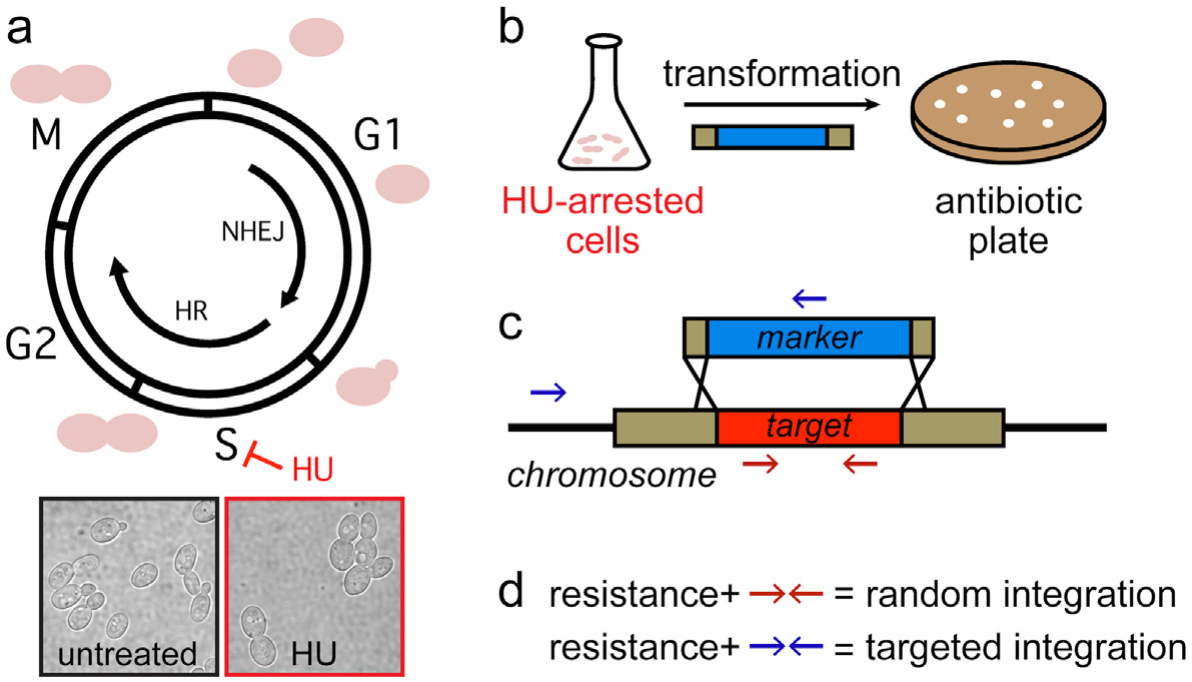
Method for increased gene targeting. Cells are grown in the presence of hydroxyurea to induce cell cycle arrest in S-phase with high HR activity (a). *Y*. *lipolytica* YB-392 cells untreated or arrested at the large-budded stage are shown. HU-arrested cells are transformed with an antibiotic resistance cassette bearing the marker flanked by short regions of homology to the promoter and terminator of the target gene (b). Homologous recombination between the cassette and genomic DNA leads to replacement of the target gene with the marker (c). Antibiotic-resistant colonies are screened by PCR to distinguish between random and targeted integration using primer sets specific to each integration outcome (d).

To test the effect of S-phase arrest on targeted integration in *Y*. *lipolytica*, actively dividing cultures of YB-392 were cultivated in the presence or absence of HU prior to transformation. Cell cycle arrest was confirmed by microscopy. An asynchronous *Y*. *lipolytica* culture presents a mixed morphology with cells at the unbudded, small-budded and large-budded stage indicating an actively dividing population. HU-treated cells are arrested at the large-budded stage (Fig 1a). Each culture was then carried through the same conventional transformation protocol to introduce a gene deletion cassette encoding a selectable marker flanked by short (37–50 bp) sequences homologous to upstream and downstream regions of the gene of interest (Fig 1b and 1c). Colonies were screened by phenotype or PCR to determine whether marker integration was random or targeted (Fig 1d). HU produced a dramatic shift in favor of targeted integration (Table 1). In agreement with our past experience with this strain, untreated YB-392 transformants contained only randomly integrated marker, rendering gene deletion unattainable. When cells were treated with HU prior to transformation, gene targeting was possible at rates that enable quick and reliable generation of knockouts. To demonstrate the reproducibility of HR stimulation, the deletion of YALI0D17534 which had the lowest level of targeting was performed three times. HU-treated cells showed targeting efficiencies of 4%, 9% and 5% whereas no targeted integration was observed without treatment in 240 colonies screened across the three experiments. We have used this method to delete 15 genes in YB-392. We have observed gene-to-gene variation from 4–96% in the efficiency of gene targeting that may be related to the local chromatin structure or phenotype of deletion (data not shown).

Given the near universal oscillatory nature of HR/NHEJ activity and widespread effectiveness of HU in cell cycle arrest, we reasoned that HU treatment would increase the rate of gene targeting in other organisms (Table 1). We found that treatment with HU increased gene targeting in the non-conventional yeast *Arxula adeninivorans* 3–8 fold depending on the target locus. To quickly evaluate the applicability of this method in three model yeast systems, we targeted the *ADE2* locus in *Pichia pastoris*, *Kluyveromyces lactis* and *Saccharomyces cerevisiae* and scored transformants for the characteristic red colony phenotype of *ade2* mutants [10, 38, 39]. In all three organisms, the percentage of red colonies increased with HU treatment prior to transformation. HU-treatment thus enabled or enhanced gene targeting in all five organisms tested. Due to the low targeting efficiency we observed at the *S*. *cerevisiae ADE2* locus, we wanted to confirm that the color phenotype is representative of the genotype. We probed a set of six white and six red colonies for the deletion-specific *ADE2*::*nat* product. Our results confirmed that all the red colonies and only the red colonies contained correct targeting of the locus.

To delete the *K*. *lactis ADE2* gene, we used the nourseothricin resistance gene (*nat*) either with or without a constitutive promoter and terminator built into the deletion cassette. In both cases, the deletion cassette was flanked by 50 base pairs of homology directly upstream and downstream of the *ADE2* gene. HU treatment increased the percentage of gene targeting with either cassette (Table 1). As we have observed in the past for *Y*. *lipolytica* (data not shown), omission of the promoter/terminator had a positive effect on gene targeting in *K*. *lactis*, presumably by restricting nourseothricin resistance to transformants where *nat* expression was driven by an endogenous promoter and terminator as would occur with correctly targeted integration at the *ADE2* locus.

To evaluate the statistical significance of our results, we calculated the hypergeometric probability of each dataset based on Fisher’s exact test [40]. This value represents the likelihood of randomly obtaining the observed distribution if the two conditions (with or without HU treatment) were equivalent. All but one value are below 3% and in the case of the least significant experiment, the first attempt at deletion of YALI0D17534, the value dropped to more significant levels when the experiment was repeated and more colonies were analyzed. An analysis of the cumulative YALI0D17534 data (16 targeted integrants per 240 colonies with HU treatment) yields a p-value of 1.2 x 10^−5^. The statistical analysis supports the conclusion that HU treatment prior to transformation enhances gene targeting in all strains and loci tested (Table 1).

## Discussion

Gene targeting is critical to the study of gene function, pathway engineering in industrial strains, and therapeutic applications. In most organisms, DNA integration after transformation is predominantly non-homologous rendering the isolation of cells with targeted integration difficult and time consuming. Random integration is the result of a high ratio of NHEJ-to-HR activity. Efforts to eliminate NHEJ through mutation have been successful but limit researchers to mutant background strains that are not available in all organisms. Furthermore, NHEJ mutants are prone to genomic instability.

We found that HU treatment prior to transformation is an effective way to synchronize cells in S-phase when HR activity is high and thus increase the rate of gene targeting. Using this treatment, we successfully increased the rate of gene targeting in five yeast strains. In the case of *Y*. *lipolytica* YB-392, HU-treatment was critical for industrial strain engineering of this host in our laboratory as it enabled gene deletions that were unattainable with standard transformation methods. Gene targeting improvements in the other strains tested were more modest, ranging from 1.2- to 8-fold. We did not optimize transformation and HU treatment protocols for these strains; it is therefore possible that higher rates could be achieved with additional work to achieve uniform cell cycle arrest and high transformation efficiency. Nonetheless, HU treatment prior to transformation consistently yielded higher rates of gene deletion underlining the wide applicability of the method.

It is important to note that in all cases gene targeting was achieved with short flanks of homology to the target locus, ranging from 37 to 50 base pairs in length (with additional homology across the initiating methionine or stop codon). Short homology regions were part of the oligonucleotide primers used to amplify the selectable markers, obviating the need to construct gene-specific plasmids with long homology regions. The ability to target genes with such short flanks expedites strain engineering and reduces associated costs. Short homology regions are also used in extrachromosomal recombination protocols such as recombination cloning and yeast-mediated ligation [41–43]. The ability to increase the rate of HR even by a small factor, could be essential in these applications.

The length of targeting homology is among several factors known to affect the efficiency of recombination [44]. In derivatives of the *S*. *cerevisiae* strain used in this study (S288C), targeting efficiency has been reported to drop from 98% to 46% when the homology length is reduced from 900 bp to 90 bp [45]. A study using 35–51 bp of homology at various chromosomal loci in *S*. *cerevisiae* strain W303-1B demonstrated targeting efficiencies ranging from 17% to 60% [46]. The short length of homology (50 bp) we used to target *ADE2* in S288C may therefore have contributed to the low (6%) targeting efficiency we observed. Low targeting efficiency could be due to a secondary effect of homology length on specificity of integration. It is reasonable that short homology regions will have partial matches at unintended loci and this would be a sequence-dependent variable. Nevertheless, any locus or sequence-dependent variables equally hold for both treated and untreated cells while the purpose of our study was to evaluate the effect of HU-treatment on the basal level of gene targeting. We observed a near-tripling of gene targeting efficiency at *S*. *cerevisiae ADE2* (from 6% to 17%) underlining the wide applicability of the method.

Other means of cell cycle synchronization are available to enrich for high HR cells. Cells can be arrested at any stage of the cell cycle using established methods (e.g. benomyl, nocodazole, α-factor, nutrient limitation [33, 47]) and released into a synchronous cell cycle from which samples can be tested for HR activity by transformation in order to identify the most efficient population. Alternatively, yeast at a specific cell cycle stage can be isolated from asynchronous cultures by elutriation. Synchronization by any of these methods does not require a specific genetic background and can be inserted as a step between growth and transformation in most transformation protocols.

As HU treatment addresses the same fundamental issue of high NHEJ-to-HR activity as the NHEJ gene deletion approach to gene targeting, it would be reasonable to expect enhanced gene targeting through HU treatment in those organisms where NHEJ gene deletion succeeded. We demonstrated that this is the case for *Y*. *lipolytica*, *K*. *lactis* and *P*. *pastoris* where NHEJ mutations were previously studied [8–10, 20]. We expect this method to be widely applicable in high NHEJ organisms, potentially including higher eukaryotes where NHEJ mutants have also been used and algae.

Biotechnology efforts often focus on non-conventional organisms that exhibit favorable characteristics with respect to the intended product and process parameters. Inability to engineer these organisms and specifically to delete genes via homologous recombination can be a barrier to their utilization. Enhancement of gene targeting through HU-mediated arrest is quick, reversible, and does not permanently alter the strain or compromise its genetic stability. The latter is especially important in maintaining the productivity, robustness and growth properties engineered in industrial strains. The method described in this study could potentially open up new microbiological industrial platforms and facilitate strain engineering in existing industrial strains.

## Methods

### Strains and media

Wild type *Yarrowia lipolytica* strain YB-392 was obtained from the ARS Culture (NRRL) Collection. The *Arxula adeninivorans* type strain (ATCC 76597), wild type *Kluyveromyces lactis* (ATCC 8585, NRRL Y-1140), wild type *Pichia pastoris* (ATCC 76273, NRRL Y-11430) and *Saccharomyces cerevisiae* (ATCC 204508, S288C) were obtained from ATCC. All strains were cultured in YPD (10 g/L yeast extract, 20g/L bacto peptone, 20 g/L glucose) with the addition of 20 g/L agar for solid media and either hygromycin B (300 μg/ml) or nourseothricin (50 μg/ml for *K*. *lactis* and *P*. *pastoris*, and 100 μg/ml for *S*. *cerevisiae*) for antibiotic selection. *Y*. *lipolytica*, *K*. *lactis*, *P*. *pastoris* and *S*. *cerevisiae* were cultured at 30°C and *A*. *adeninivorans* at 37°C. Hydroxyurea (HU) was obtained from Sigma-Aldrich. *Y*. *lipolytica* YALI0B13970 deletion candidates were screened on minimal media (6.7 g/L Yeast Nigrogen Base without amino acids, 20 g/L agar) supplemented with 20 g/L glucose or glycerol.

### Generation of deletion cassettes

The *E*. *coli hph* gene, conferring hygromycin B resistance (GenBank: AEJ60084.1) and the *Streptomyces noursei nat* gene, conferring nourseothricin resistance (GenBank: CAA51674.1) were codon-optimized for expression in *Y*. *lipolytica* (Genscript) (S1 Text). For deletions in *Y*. *lipolytica* and *A*. *adeninivorans*, the *hph* gene was amplified by PCR using outer primers that attach short flanks homologous to the promoter and terminator of target genes immediately 5’ and 3’ to the ORF in combination with internal *hph* primers. A two-fragment deletion cassette was thus made for each target such that the fragments overlapped in the *hph* reading frame but neither fragment alone contained the entire functional gene. Omission of a full promoter and terminator in the cassette and splitting of the marker coding sequence into two PCR fragments reduce the probability that random integration of these pieces will be selected [48]. Marker integration at the target locus by homologous recombination enhances transcription using the intact local promoter and terminator. For deletions in *P*. *pastoris*, *K*. *lactis* and *S*.*cerevisiae*, the *nat* gene was amplified in two overlapping fragments with outer primers that provide homology 5’ and 3’ of the *ADE2* gene as described for *hph* constructs. In addition to this deletion cassette, a cassette that included a promoter and terminator for *nat* expression was used to target the *K*. *lactis ADE2* gene. The target homology regions were the same as for the deletion cassette without a promoter/terminator. The two PCR products for each target gene were co-transformed into *Y*. *lipolytica* without purification. The PCR products were purified using a PCR purification kit (Qiagen) and co-transformed into *A*. *adeninivorans*, *P*. *pastoris*, *K*. *lactis* and *S*. *cerevisiae*.

The *Y*. *lipolytica* [49], *P*. *pastoris*, *K*. *lactis* and *S*. *cerevisiae* genomes are publicly available. The genome of *A*. *adeninivorans* strain ATCC 76597 was sequenced by Synthetic Genomics for Novogy Inc. and used to design deletion cassettes for this study. The relevant sequences are accessible at GenBank (#KM409710-4). A genome for *A*. *adeninivorans* has also recently been published [50]. All primers used to amplify deletion cassettes in this study are listed in (S1 and S3 Tables).

### HU arrest

*Y*. *lipolytica* grown overnight on solid YPD media was used to inoculate a 25-ml YPD culture at OD_600_ = 0.5. After 3 hrs of growth, 50 mM HU was added. Cells were harvested 2 hrs post HU addition. HU treatment was determined to arrest growth while maintaining viability of the cells (data not shown) [51–53]. *A*. *adeninivorans*, *P*. *pastoris*, *K*. *lactis* and *S*. *cerevisiae* grown overnight in liquid YPD cultures were diluted 1:10 in 25-ml YPD. After 3.5 hrs of growth, HU was added to 25 mM (*A*. *adeninivorans*), 100 mM (*K*. *lactis*) and 200 mM (*S*. *cerevisiae* and *P*. *pastoris*). Cells were harvested 2 hrs post HU addition.

### Transformation

*Y*. *lipolytica* cells were washed with water and resuspended in a pellet volume of water. 50 μl was aliquoted per transformation reaction, cells were centrifuged and the supernatant was discarded. 9 μl of each PCR product (without purification) and 92 μl of transformation mix (80 μl 60% PEG4000, 5 μl 2M DTT, 5 μl 2M lithium acetate pH 6, 2 μl 10 mg/ml single stranded salmon sperm DNA) were added to the cell pellet. The transformation reaction was mixed well by vortexing and heat shocked at 39°C for 1 hr. Cells were centrifuged, the supernatant was discarded, cells were resuspended in 1 ml YPD, transferred to culture tubes and cultured overnight before plating on selective media.

*A*. *adeninivorans*, *P*. *pastoris*, *K*. *lactis* and *S*. *cerevisiae* cells were washed with water and resuspended in 2 ml 100 mM lithium acetate and 40 μl 2 M DTT. The mixture was incubated at 37°C (*A*. *adeninivorans*) or 30°C (*P*. *pastoris*, *K*. *lactis* and *S*. *cerevisiae*) with rotation for 1 hr. From this point on, cells and reagents were kept on ice. Cells were harvested by centrifugation, washed with water followed by 1 M sorbitol and resuspended in 2 ml 1 M sorbitol. 40 μl of this suspension and 2.5 μl of each purified PCR product were mixed in a chilled 0.2 cm electroporation cuvette per transformation reaction. Electroporation was performed at 25 μF, 200 Ω, 1.5 kV. For *P*. *pastoris*, *K*. *lactis* and *S*. *cerevisiae*, 1 ml of 1 M sorbitol was added to the cuvettes after electorporation and cells were incubated for 1 hr at 30°C. Cells were transfered to culture tubes containing 1 ml YPD and cultured overnight before plating on selective media.

### Transformant screening

For PCR-based screening, antibiotic-resistant colonies were patched onto fresh plates to confirm antibiotic resistance. A small amount of cells from each patch was picked and smeared into PCR tubes, microwaved for 2 min and then frozen at -20°C for 5 min before being used as the template for PCR analysis with primer sets specific to random or targeted integration outcomes (Fig 1, S2 and S3 Tables). Results were scored and presented in Table 1 as gene targeting percentage of total transformants screened. *Y*. *lipolytica* YALI0B13970 (*GUT2*, mitochondrial glycerol-3-phosphate dehydrogenase) deletion leads to inability to utilize glycerol as the sole carbon source. Transformants were therefore screened for lack of growth on media containing glycerol as the sole carbon source and the loss of the YALI0B13970 gene was confirmed by the absence of the gene-specific product. *P*. *pastoris*, *K*. *lactis* and *S*. *cerevisiae ade2* deletion produces pink colonies. Transformants were therefore scored visually and are presented in Table 1 as the percentage of red colonies out of the total number of colonies screened. Targeted integration was also probed by PCR for the deletion-specific *ADE2*::*nat* product in a subset of 6 red and 6 white *S*. *cerevisiae* transformants.

### Fisher’s exact test

The exact hypergeometric probability of obtaining each experimental result in Table 1 was determined using the Fisher’s exact test formula:

$$p\;=\;\frac{(a+b)!(c+d)!(a+c)!(b+d)!}{a!b!c!d!n!}$$

where a is the number of targeted integrations observed in untreated cells, b is the number of targeted integrations observed in treated cells, c is the number of random integrations observed in untreated cells, d is the number of random integrations observed in treated cells and n is the sum of all observations (a+b+c+d).

## Supporting Information

S1 Text Sequences used in deletion cassettes. (DOCX)

S1 Table Primer pairs used to construct deletion cassettes. (DOCX)

S2 Table Primer sets used for PCR analysis of transformants. (DOCX)

S3 Table Primer sequences. (DOCX)
