# Supplementary material for: Improved Gene Targeting through Cell Cycle Synchronization
Source: PLoS One. 2015 Jul 20;10(7):e0133434. doi: 10.1371/journal.pone.0133434 (PMC4507847; doi:10.1371/journal.pone.0133434)
Supplement: S1 Text — (DOCX) [file pone.0133434.s001.docx]

**S1 Text. Sequences used in deletion cassettes.**

*hph* gene codon-optimized for *Y. lipolytica* expression

ATGAAGAAGCCCGAGCTGACCGCTACCTCTGTTGAGAAGTTCCTGATTGAGAAGTTTGATTCCGTTTCCGACCTGATGCAGCTGTCCGAGGGCGAGGAGTCTCGAGCCTTCTCCTTTGACGTGGGCGGACGAGGTTACGTTCTGCGAGTGAACTCGTGTGCCGACGGCTTCTACAAGGATCGATACGTCTACCGACACTTTGCTTCTGCCGCTCTGCCCATCCCTGAGGTTCTCGACATTGGCGAGTTCTCTGAGTCCCTCACCTACTGCATCTCTCGACGAGCTCAGGGAGTCACCCTGCAGGACCTCCCTGAGACTGAGCTGCCTGCTGTCCTCCAGCCTGTTGCTGAGGCCATGGACGCTATCGCTGCTGCTGATCTGTCCCAGACCTCGGGTTTCGGCCCCTTTGGACCTCAGGGAATTGGACAGTACACCACTTGGCGAGACTTCATCTGTGCTATTGCCGATCCTCACGTCTACCATTGGCAGACCGTTATGGACGATACTGTGTCGGCTTCTGTCGCTCAGGCTCTGGACGAGCTGATGCTCTGGGCCGAGGATTGCCCCGAGGTTCGACACCTGGTGCATGCTGACTTCGGTTCCAACAACGTTCTCACCGACAACGGCCGAATCACTGCCGTGATTGACTGGTCCGAGGCTATGTTTGGCGACTCGCAGTACGAGGTGGCCAACATCTTCTTTTGGCGACCCTGGCTGGCTTGTATGGAGCAGCAGACCCGATACTTCGAGCGACGACATCCTGAGCTCGCTGGATCCCCTCGACTGCGAGCTTACATGCTCCGAATTGGTCTGGACCAGCTCTACCAGTCGCTGGTGGATGGCAACTTTGACGATGCTGCCTGGGCTCAGGGACGATGTGACGCCATCGTGCGATCTGGCGCTGGAACCGTCGGACGAACTCAGATTGCCCGACGATCCGCTGCTGTCTGGACCGACGGATGCGTGGAGGTCCTGGCTGATTCGGGTAACCGACGACCCTCTACTCGACCTCGAGCTAAGGAGTAA

*nat* gene codon-optimized for *Y. lipolytica* expression

ATGACCACTCTGGATGACACCGCTTACCGATACCGAACTTCCGTTCCTGGCGATGCCGAGGCTATTGAGGCTCTGGATGGATCTTTCACCACTGACACCGTTTTCCGAGTGACCGCTACTGGCGACGGCTTCACCCTGCGAGAGGTGCCTGTCGACCCTCCTCTCACCAAGGTTTTCCCTGACGATGAGTCGGACGATGAGTCTGACGCTGGAGAGGACGGCGACCCTGACTCTCGAACTTTCGTGGCTTACGGCGACGATGGAGACCTGGCCGGCTTTGTGGTCGTTTCTTACTCCGGATGGAACCGACGACTGACCGTGGAGGACATCGAGGTCGCTCCTGAGCACCGAGGTCATGGTGTCGGACGAGCTCTGATGGGTCTCGCTACTGAGTTCGCTCGAGAGCGAGGTGCTGGCCACCTGTGGCTCGAGGTCACCAACGTTAACGCCCCTGCTATTCATGCCTACCGACGAATGGGTTTTACCCTGTGTGGCCTCGATACTGCCCTGTACGACGGAACCGCTTCCGATGGAGAGCAGGCCCTCTACATGTCGATGCCCTGCCCTTAA

*nat* gene expression cassette including *S. cerevisiae* Tef1 promoter and *S. cerevisiae* Cyc1 terminator

ATAGCTTCAAAATGTTTCTACTCCTTTTTTACTCTTCCAGATTTTCTCGGACTCCGCGCATCGCCGTACCACTTCAAAACACCCAAGCACAGCATACTAAATTTCCCCTCTTTCTTCCTCTAGGGTGTCGTTAATTACCCGTACTAAAGGTTTGGAAAAGAAAAAAGAGACCGCCTCGTTTCTTTTTCTTCGTCGAAAAAGGCAATAAAAATTTTTATCACGTTTCTTTTTCTTGAAAATTTTTTTTTTTGATTTTTTTCTCTTTCGATGACCTCCCATTGATATTTAAGTTAATAAACGGTCTTCAATTTCTCAAGTTTCAGTTTCATTTTTCTTGTTCTATTACAACTTTTTTTACTTCTTGCTCATTAGAAAGAAAGCATAGCAATCTAATCTAAGTTTTAATTACAAAATGACCACTCTGGATGACACCGCTTACCGATACCGAACTTCCGTTCCTGGCGATGCCGAGGCTATTGAGGCTCTGGATGGATCTTTCACCACTGACACCGTTTTCCGAGTGACCGCTACTGGCGACGGCTTCACCCTGCGAGAGGTGCCTGTCGACCCTCCTCTCACCAAGGTTTTCCCTGACGATGAGTCGGACGATGAGTCTGACGCTGGAGAGGACGGCGACCCTGACTCTCGAACTTTCGTGGCTTACGGCGACGATGGAGACCTGGCCGGCTTTGTGGTCGTTTCTTACTCCGGATGGAACCGACGACTGACCGTGGAGGACATCGAGGTCGCTCCTGAGCACCGAGGTCATGGTGTCGGACGAGCTCTGATGGGTCTCGCTACTGAGTTCGCTCGAGAGCGAGGTGCTGGCCACCTGTGGCTCGAGGTCACCAACGTTAACGCCCCTGCTATTCATGCCTACCGACGAATGGGTTTTACCCTGTGTGGCCTCGATACTGCCCTGTACGACGGAACCGCTTCCGATGGAGAGCAGGCCCTCTACATGTCGATGCCCTGCCCTTAAACAGGCCCCTTTTCCTTTGTCGATATCATGTAATTAGTTATGTCACGCTTACATTCACGCCCTCCTCCCACATCCGCTCTAACCGAAAAGGAAGGAGTTAGACAACCTGAAGTCTAGGTCCCTATTTATTTTTTTTAATAGTTATGTTAGTATTAAGAACGTTATTTATATTTCAAATTTTTCTTTTTTTTCTGTACAAACGCGTGTACGCATGTAACATTATACTGAAAACCTTGCTTGAGAAGGTTTTGGGACGCTCGAAGGCTTTAATTTGC
