# Supplementary material for: Improved Gene Targeting through Cell Cycle Synchronization
Source: PLoS One. 2015 Jul 20;10(7):e0133434. doi: 10.1371/journal.pone.0133434 (PMC4507847; doi:10.1371/journal.pone.0133434)
Supplement: S1 Table — (DOCX) [file pone.0133434.s002.docx]

**S1 Table. Primer pairs used to construct deletion cassettes.**

| gene deletion target | 5’ fragment | | 3’ fragment | |
| --- | --- | --- | --- | --- |
|  | forward | reverse | forward | reverse |
| YALI0D17534 | NP1798 | NP656 | NP655 | NP1799 |
| YALI0B13970 | NP1563 | NP656 | NP655 | NP1800 |
| KM409710 | NP2197 | NP656 | NP655 | NP2198 |
| KM409711 | NP2199 | NP656 | NP655 | NP2200 |
| KM409712 | NP2201 | NP656 | NP655 | NP2202 |
| KM409713 | NP1935 | NP656 | NP655 | NP1936 |
| KM409714 | NP2203 | NP656 | NP655 | NP2204 |
| KLLA0E02685 *Kl ADE2* (with promoter/terminator) | NP2440 | NP356 | NP355 | NP2441 |
| KLLA0E02685 *Kl ADE2* | NP2442 | NP356 | NP355 | NP2443 |
| PAS_chr3_0085 *Pp ADE2* | NP2446 | NP356 | NP355 | NP2447 |
| YOR128C *Sc ADE2* | NP2450 | NP356 | NP355 | NP2451 |
