# Supplementary material for: Improved Gene Targeting through Cell Cycle Synchronization
Source: PLoS One. 2015 Jul 20;10(7):e0133434. doi: 10.1371/journal.pone.0133434 (PMC4507847; doi:10.1371/journal.pone.0133434)
Supplement: S2 Table — (DOCX) [file pone.0133434.s003.docx]

**S2 Table. Primer sets used for PCR analysis of transformants.**

| Gene deletion target | Random integration | | Targeted integration | |
| --- | --- | --- | --- | --- |
|  | Internal forward | Internal reverse | External forward | Marker reverse |
| YALI0D17534 |  |  | NP1033 | NP656 |
| YALI0B13970 | NP1495 | NP1499 |  |  |
| KM409710 | NP2206 | NP2207 | NP2205 | NP656 |
| KM409711 | NP2209 | NP2210 | NP2208 | NP656 |
| KM409712 | NP2212 | NP2213 | NP2211 | NP656 |
| KM409713 | NP2146 | NP2147 | NP2145 | NP656 |
| KM409714 | NP2215 | NP2216 | NP2214 | NP656 |
| YOR128C |  |  | NP2430 | NP356 |
