# Supplementary material for: Improved Gene Targeting through Cell Cycle Synchronization
Source: PLoS One. 2015 Jul 20;10(7):e0133434. doi: 10.1371/journal.pone.0133434 (PMC4507847; doi:10.1371/journal.pone.0133434)
Supplement: S3 Table — (DOCX) [file pone.0133434.s004.docx]

**S3 Table. Primer sequences (*hph* or *nat* annealing sequences underlined).**

| NP355 | AACTTCCGTTCCTGGCGATGCCG |
| --- | --- |
| NP356 | TCTCCATCGGAAGCGGTTCCGTC |
| NP655 | ACCACTTGGCGAGACTTCATCTGT |
| NP656 | AGCATCGTCAAAGTTGCCATCCAC |
| NP1033 | CCTCCGGTCGATTATGCCTC |
| NP1495 | CGTTCGTCTGGTCTGCTCAC |
| NP1499 | TAGCTCCTCCACCAACAACG |
| NP1563 | TCAATGTAGACGTCGCGGAACTTCTCTTTCCTACCCACCACCCCAAACAAATGAAGAAGCCCGAGCTGACC |
| NP1798 | CAGCTCTCTTCCCCCGTTCAGCTCCTTTTCTACCGCGATTATGAAGAAGCCCGAGCTGAC |
| NP1799 | TTTAGTCTCATCGTTAGTAGTTATGTGCTCTGCTCGGGGTTACTCCTTAGCTCGAGGTCG |
| NP1800 | CAAGTGATTATCTTATTGGCTATACGCTTTTACTATACAGTTACTCCTTAGCTCGAGGTCG |
| NP1935 | CGGCCTGTAGCCTCGAAAGCTAACATACATTATCAAGTGATGAAGAAGCCCGAGCTGACC |
| NP1936 | TCTGGAGACAACGTCAAGTTTTTCTATCGCTGAAACTCGATTACTCCTTAGCTCGAGGTCGAG |
| NP2145 | CTCAGTCGGTAGAGCGTTCG |
| NP2146 | TCTGGACGGAGAAGGTGTGG |
| NP2147 | AACAAGGACCCGCCGTGAAG |
| NP2197 | AGGACAAGTGTTCAACTAGTCTTCAACTGGTTCTTTGCTATGAAGAAGCCCGAGCTGACC |
| NP2198 | AAACAAAACAAATCAATTCAAACAAAACAAACTACCTTTACTCCTTAGCTCGAGGTCGAG |
| NP2199 | CATTGCGCGAGACTTTTAGTCGACTCCAACTGGATCACAATGAAGAAGCCCGAGCTGACC |
| NP2200 | AATATAATCTAATGAATGATAGGTCGAATGCTTGTTATTACTCCTTAGCTCGAGGTCGAG |
| NP2201 | GGTTCTATTTTATCACAAAACCTTCGATAATATAACACAATGAAGAAGCCCGAGCTGACC |
| NP2202 | AGGATGATGTCCCAAGTACGTACGTCGTTAGATAATGCAATTACTCCTTAGCTCGAGGTCGAG |
| NP2203 | CAACTGTAGAAGCTTTGAGACACAGTCCAACGGGTAGGAATGAAGAAGCCCGAGCTGACC |
| NP2204 | TTTAAAGCTGTCTAATTGCCATGAATGAAGACCGTTGCGGTTACTCCTTAGCTCGAGGTCGAG |
| NP2205 | GAGCCTTAAACCGCAGCACC |
| NP2206 | TATGACGGCTCCCAAGGAGG |
| NP2207 | AGTTGGAGTCATCGCGCTGG |
| NP2208 | TGGTAACCGGACCTGGCAAG |
| NP2209 | TGTGGTGGCTCGGTTACTGC |
| NP2210 | TGCTCTTGACATGGCTGTCTGC |
| NP2211 | AGCCGCACACTTTCACGAGC |
| NP2212 | ACTCCTCACGTTGCTGCCAC |
| NP2213 | AACCCTGGCCGAATCCGTTG |
| NP2214 | ACAGCGGAAGGCAAGGATGG |
| NP2215 | TTGGACGAACAGCCCTGGTG |
| NP2216 | ACGATGCACACACTGCCGAC |
| NP2430 | TCGCCCAATGTGTCCATCTG |
| NP2440 | TTGATAACCCTATATAATAACATCACGTTCACCTTCTTCACTAAGACAACATAGCTTCAAAATGTTTCTACTCC |
| NP2441 | GATCGAAACAGGAGTAGTAAGATTATACACGATATAAAGGGTTTTCTGACGCAAATTAAAGCCTTCGAGC |
| NP2442 | TTGATAACCCTATATAATAACATCACGTTCACCTTCTTCACTAAGACAACATGACCACTCTGGATGACACC |
| NP2443 | GATCGAAACAGGAGTAGTAAGATTATACACGATATAAAGGGTTTTCTGACTTAAGGGCAGGGCATCGACA |
| NP2446 | GTTACCTTTTCCAAGAATCGTAGAAACGATTAAAAAACTTCCAAACTCTCATGACCACTCTGGATGACACC |
| NP2447 | TAAGTTACGTGTACTATTATATATACATCTATTAAAAAACAATACTAAATTTAAGGGCAGGGCATCGACA |
| NP2450 | CCTACTATAACAATCAAGAAAAACAAGAAAATCGGACAAAACAATCAAGTATGACCACTCTGGATGACACC |
| NP2451 | ATATCATTTTATAATTATTTGCTGTACAAGTATATCAATAAACTTATATATTAAGGGCAGGGCATCGACA |
